# Molecular typing methods to characterize *Brucella* spp. from animals: A review

**DOI:** 10.14202/vetworld.2024.1778-1788

**Published:** 2024-08-13

**Authors:** Aida Daugaliyeva, Saule Daugaliyeva, Nazerke Kydyr, Simone Peletto

**Affiliations:** 1 LLP “Kazakh Research Institute for Livestock and Fodder Production,” St. Zhandosova 51, Almaty 050035, Kazakhstan; 2 LLP “Scientific Production Center of Microbiology and Virology,” Bogenbay Batyr Str. 105, Almaty 050010, Kazakhstan; 3 Experimental Zooprofilactic Institute of Piedmont, Liguria and Aosta Valley, Via Bologna 148, 10154 Turin, Italy

**Keywords:** *Brucella*, molecular typing, multilocus sequence typing, multilocus variable-number tandem-repeat analysis, single-nucleotide polymorphisms, whole-genome sequencing

## Abstract

Brucellosis is an infectious disease of animals that can infect humans. The disease causes significant economic losses and threatens human health. A timely and accurate disease diagnosis plays a vital role in the identification of brucellosis. In addition to traditional diagnostic methods, molecular methods allow diagnosis and typing of the causative agent of brucellosis. This review will discuss various methods, such as Bruce-ladder, Suiladder, high-resolution melt analysis, restriction fragment length polymorphism, multilocus sequence typing, multilocus variable-number tandem repeat analysis, and whole-genome sequencing single-nucleotide polymorphism, for the molecular typing of *Brucella* and discuss their advantages and disadvantages.

## Introduction

Brucellosis is an infectious zoonotic disease of livestock and wild animals. At present, it is widespread mainly in developing countries of Central Asia and in countries of the European Mediterranean Basin, with some populations in North Africa and the Middle East where the incidence rates are highest [1, 2], over 250/100,000 [3]. This disease significantly impacts national economies by reducing livestock productivity [4–11]. Approximately 500,000 annual human infections worldwide make brucellosis the most common zoonotic disease. The true human incidence of tuberculosis, according to the World Health Organization (WHO), is 25 times greater than the reported cases [12, 13]. Brucellosis is reported to affect over 10 in 100,000 people in endemic areas [14]. Brucellosis is contracted from aborted fetuses and placentas, unpasteurized milk and dairy, aerosols, and injuries to the skin and mucous membranes [5, 15].

Acute form is the most common manifestation of the infection [16]. It is characterized by undulating fever (Maltese fever) [5], sweating, weakness, fatigue, anorexia, weight loss, headache, and general aching pain [17]. This condition can progress to a chronic disease in rare cases with severe cardiac (endocarditis), neurological (personality changes, meningitis, encephalitis, peripheral neuropathy), or visceral complications (hepatosplenomegaly) [18–20]. The disease’s clinical signs vary depending on the genomic makeup of the pathogen [21]. Pregnant women and animals experience miscarriages and premature births. These conditions in males can cause orchitis and epididymitis can lead to infertility [22]. *Brucella* exhibits varying degrees of virulence. *Brucella melitensis* causes the most severe human disease, while *Brucella suis* follows in the second place. The less virulent for humans of the five biovars in the *B. suis* species is biovar 2. People with weakened immune systems are susceptible to brucellosis caused by *B. suis* biovar 2.

Antibiotics are used to treat acute Brucellosis infections in humans. In the chronic phase, the bacteria evade immune response and antibiotic treatment due to their intracellular location. Livestock with brucellosis cannot be treated.

Brucellosis is caused by a potential biological warfare agent classified under category B [1]. Due to its transmission through air and food [23], it can infect both humans and animals. Brucellosis’s early symptoms resemble those of flu. To rule out biological terrorism, it is necessary to determine the origin of infectious agents through genotyping.

The control of brucellosis requires careful surveillance and highly discriminatory methods of strain characterization, investigation of the elements of the epizootic chain, identification of the source of the pathogen, and monitoring of the transmission mechanism [24]. Epidemiologic studies use bio- and genotyping approaches to identify circulating *Brucella* species, genotypes, and biovar-causing outbreaks. Identifying *Brucella* species, biovars, and genotypes in specific infection areas is crucial for classifying foci, evaluating epidemiological intensity, clarifying the reservoir and source, tracing transmission, and determining effective treatment strategies. Various factors, including unsanitary conditions on farms, economic circumstances in the country, consumption of raw animal products, human awareness levels, climatic conditions, and environmental hygiene influence the emergence and circulation of pathogens.

The World Organization for Animal Health (WOAH) advises using biochemical and serological tests, phenotyping, and host specificity for culture growth when investigating brucellosis pathogens [25, 26]. These approaches, which involve handling live pathogens and tracing outbreak sources, are dangerous and labor-intensive [27–30]. To conduct bacteriological tests, you need a BSL-3 laboratory and qualified personnel who can handle live pathogens [31, 32]. Isolating a pure culture is definitive proof for diagnosing brucellosis. Advanced scientific techniques, such as serological reactions, bacteriological studies, and molecular analysis, are necessary to fully understand the agents responsible for brucellosis.

This review assesses *Brucella’s* molecular typing techniques, detailing their respective benefits and drawbacks. This review explores some molecular techniques. *Brucella* species and vaccine strains are identified using multiplex polymerase chain reaction (PCR) through Bruce-ladder. DNA fragments are analyzed by gel electrophoresis after being cut using restriction endonucleases in the restriction fragment length polymorphism (RFLP) method. High-resolution melting analysis (HRM) identifies mutations and polymorphisms within DNA sequences. Multilocus sequence typing (MLST) identifies microbial strains by sequencing selected gene segments. Multiple locus variable-number tandem repeat analysis (MLVA) involves analyzing the polymorphisms in tandemly repeated DNA sequences of pathogenic bacteria. Polymorphism identifies a one-nucleotide difference in the size of a DNA sequence. Whole-genome sequencing (WGS) reveals the complete DNA sequence of an organism’s genome.

## Phenotype and Genotype Characteristics of the Causative Agent of Brucellosis

*Brucella*, a Gram-negative proteobacterium and facultative intracellular, non-spore-forming bacterium of the genus *Brucella*, is the causative agent of brucellosis [9, 22, 33]. The *Brucella* genus harbors relatively stable bacteria with minor genetic disparities [9]. *Brucella* displays unique traits specific to its species and biovar. The phenotype is identified through phenotypic methods. A phenotype represents an individual’s unique combination of observable traits, formed through the interaction of genes and environmental influences during development. 14 species were identified based on shared biochemical traits and host preferences (Table-1) [17, 31, 34–41].

**Table-1:** Classification of *Brucella* species.

*Brucella* species	Primary host	Biovar	Secondary hosts	References
*Brucella melitensis*	Sheep and goats	1–3	Humans, camels, dogs, pigs, and cattle	[37–39]
*Brucella abortus*	Cattle (buffalo, elk, bison)	1–6, 9	Humans, sheep, goats, wild animals, dogs, cats, camels	
*Brucella suis*	Pigs	1–3	Humans	
	Wild boars and hares	2	Humans, sheep, goats, dogs	
	Reindeer, caribou	4	Humans	
	Rodent	5	Humans	
*Brucella ovis*	Sheep			
*Brucella neotomae*	Desert wood rats		Humans	
*Brucella canis*	Dog		Humans	
*Brucella ceti*	Cetaceans		Humans	
*Brucella pinnipedialis*	Seal		Humans	
*Brucella microti*	Field voles, foxes, and soil			
*Brucella inopinata*	Human			
*Brucella papionis*	Baboons			
*Brucella vulpis*	Red fox			
*Brucella amazoniensis*	Human			[40]
*Brucella nosferati*	Bats			[41]

It has been proposed that closely related species of *Ochrobactrum* should be categorized as *Brucella* [42, 43]. Atypical species of *Brucellae* have diversified the genus of pathogens [35, 44, 45]. Motile *Brucella* spp. was isolated from amphibians, although the genus historically consisted of sessile species [45–47].

Most human infections are caused by species, such as *B. melitensis*, *Brucella abortus*, *B. suis*, and sometimes, *Brucella canis* [5, 9, 48, 49]. *B. melitensis* is a common and virulent *Brucella* species [50, 51], and it is sometimes fatal [5, 50, 52, 53]. Severe diseases in humans are also caused by *B. suis* (except biovar two) [15, 54]. Humans are also at high risk of contracting brucellosis from consuming raw milk from cows contaminated with *B. melitensis*.

In addition to infection of primary hosts, cross-infection of secondary animal hosts with *Brucella* is also possible [29, 30, 55]. *B. melitensis* also infects dogs, pigs, camels, and wild animals [26, 56, 57]. Cross-infection between animals has been observed on farms where cattle, sheep, and goats reside [56, 58–60].

To identify and differentiate species and biovar of the genus *Brucella* in 1972, the FAO/WHO Expert Committee established special tests: Increased content: CO_2_, phage lysis: Tb, H_2_S release, growth pattern on nutrient media with aniline dyes: thionin (1:25,000–1:100,000) and (1:50,000–1:100,000) fuchsin, then agglutination with monospecific sera “A” and “M” and antisera: S- and R-, in addition, growth on nutritional environments with substrates that include individual carbohydrates, urea, and amino acids.

*Brucella* biovar was isolated by studying the DNA nucleotide sequence of outer membrane proteins (OMPs). The OMP *Brucella* spp. were identified in the early 1980s and characterized as potential immunogenic and protective antigens. A previous study has identified omp31 and BP26 as candidate antigens with high potential for clinical diagnosis of brucellosis [61]. The antigens Omp22, Omp25, and Omp31 are essential proteins of *B. melitensis*, the absence of which in mutant species reduces their pathogenicity; therefore, these proteins are critical factors in bacterial pathogenicity [62]. The species *B. melitensis i*s represented by three biovar: Sheep, goats, and camels.

Five *B. suis* biovars, which are the primary carriers among pigs, represent the species. *B. suis* biovar 2 infections differ from those caused by biovar 1 and 3 in terms of host preference, location, and virulence. Few cases of human brucellosis have previously involved biovar 2. Hunters with reduced immunity and dogs in Australia, the USA, and France have been identified as sources of *B. suis* biovar 2 infection. Dogs can become infected during hunting by contacting wild pigs or their meat [63]. Occasionally, asymptomatic *B. suis* biovar 2 infections have been reported in sheep and goats that came into contact with infected wild boar.

*B. canis* results in reproductive issues and non-specific lameness in dogs. The risk of *B. canis* infection in humans is minimal, mostly affecting veterinary personnel and dog owners with compromised immune systems. The strain’s lack of surface O-polysaccharide causes this phenomenon [64]. The complex epidemiological scenario can make brucellosis monitoring and management challenging [65]. The advantage of phenotypic methods is that they allow for an accurate understanding of the interspecific relationships of the genus *Brucella*, which is necessary for understanding the epidemiology of the disease [66]. Working with a live pathogen poses a disadvantage.

The *Brucella* genome comprises two circular chromosomes, one measuring 1.2 Mb and the other 2.1 Mb [67, 68]. *B. suis* biovar 3 possesses a 3.1 Mb chromosome. Bacterial chromosomes are characterized by the replication of large chromosomes (Chp I), while plasmids exhibit the replication of smaller chromosomes (Chp II). Chr I harbored the most significant genes. This is consistent with other authors who noted that *B. abortus* and *B. melitensis* are more closely related, in contrast to *B. abortus* and *B. suis* [67]. *B. suis* is more similar to *B. abortus* than *B. melitensis*.

## Methods for Genotyping *Brucella*

Molecular typing methods include Bruce-ladder, Suis-ladder, HRM, RFLP, MLST, MLVA, and WGS single-nucleotide polymorphism (WGS-SNP).

### Bruce-ladder and Suis-ladder methods

The WOAH recommends using a Bruce-ladder based on classical PCR to diagnose brucellosis and identify and type *Brucella* species. The method involves using eight primer pairs in one reaction.

The proposed method has the following advantages: it is cheap and fast. The advantage of this method is its ability to distinguish infected animals from those vaccinated with strains S19, RB51, and Rev. 1. PCR with *B. abortus* S19 DNA does not produce a 587-bp fragment common to all *Brucella* strains tested, and the absence of a 1682-bp fragment distinguishes *B. abortus* RB51 and 1320 bp, as well as a specific additional fragment of 2524 bp. The B. melitensis Rev. 1 vaccine strain was readily distinguished from other B. melitensis strains by a specific additional 218-bp fragment. In 2011, Lopez-Goñi *et al*. [69] published an advancement of the original Bruce-ladder PCR protocol, which allows the correct discrimination of *Brucella* species, including *Brucella microti*, *Brucella inopinata*, *Brucella ceti*, *Brucella pinnipedialis*, *B. suis*, and *B. canis*. The Suis-ladder method was developed to improve the Bruce-ladder, which genotypes field strains of *B. suis* to biovar. The method also discriminates against closely related species such as *B. suis*, *B. canis*, and *B. microti* [69].

The disadvantages of PCR-based methods include cross-contamination and time-consuming electrophoresis. A more advanced method is real-time PCR, which avoids contamination because the tubes do not need to be opened to obtain results.

### HRM

A practical method for differentiating *Brucella* species is HRM, which is performed after real-time PCR. In this method, the melting curves of the amplicons are compared to detect differences in nucleotides. This analysis allowed us to immediately distinguish five species of *Brucella* in one test tube: *B. abortus*, *B. melitensis*, *B. ovis*, *B. suis*, and *B. canis* using SNP markers.

The method’s advantages include the ability to distinguish, in addition to the five species of *Brucella*, *B. microti*, *B. ceti*, *B. pinnipedialis*, and vaccine strains of *B. abortus*. Other researchers have increased the power of HRM by differentiating *B. suis* into biovar 1, 2, and 3 and identifying the vaccine strain *B. melitensis* Rev.1 [70]. The disadvantage of this method is that it can only detect SNPs that change the guanine and cytosine (GC)% fragment [71].

### RFLP

This method for genotyping *Brucella* species and biovar is based on PCR, which involves processing amplicons with restriction enzymes. Specific bands can be visualized by gel electrophoresis.

It is inexpensive and allows the identification or differentiation of strains. The disadvantages of the PCR-RFLP method include its slowness, labor intensity (it can take a month), and lack of discriminatory power. The RFLP method demonstrates different results in different laboratories and even in the same laboratory performed by other specialists.

## MLST and a core genome MLST (cgMLST) analysis

MLST typing based on multilocus sequences is a method of genetic typing of organisms based on determining the nucleotide sequence of a particular set of genes (loci). This method was first proposed in 1998 for rapid and reliable typing of pathogenic bacteria.

The progress of molecular genetic techniques has helped scientists understand the structure and differences of brucellosis pathogens in different countries. In particular, MLST and MLVA are used to investigate the geographical origins of strains and their genetic relationships. Gene comparison using cgMLST and SNP analysis has partially replaced existing biotyping methods [23, 50, 53].

### MLST

Surveillance tools such as MLST have been used to type *Brucella* to investigate the causes of disease emergence. Previously, MLST included the investigation of 9 loci, and then, 12 loci were added [72] and it was found that the *B. abortus* species consists of 3 clades (A, B, C), where clades A and B belong to African strains. In contrast, clade C (C1 and C2) is distributed worldwide [73, 74]. Clade A included strains from Mozambique and Kenya [72]. The A and B clades within *B. abortus* suggest that *B. abortus* may have spread to/from South Africa because of socioeconomic, migratory relations between countries [74].

Examples of MLST typing showed that the Egyptian isolates of *B. abortus* and *B. melitensis* were genetically unique compared with publicly available global strain sequences: *B. melitensis* was identified as sequence type (ST) 11 and *B. abortus* as ST1 [9]. *B. melitensis* isolates from India and China were identified as ST8 and were highly similar to isolates circulating on the Asian continent [25]. The most common sequence types in Iran were *B. abortus* ST1 and ST2 [9, 75, 76] and *B. melitensis* ST8, in addition to ST7 and ST10 [77]. The predominant *Brucella* species is *B. melitensis* biovar 1 [78].

#### Advantages of the MLST method

- Helps determine the geographic origin and distribution of strains [79, 80]
- Helps determine the genetic relationships among strains
- Helps predict which genotypes will prevail in the future [9]
- Helps conduct evolutionary research.

#### Disadvantages of the MLST method

- MLST cannot wholly distinguish isolates because it does not examine the entire genome of the pathogen [81]
- Does not determine the exact origin of the strains at the outbreak site or the transmission route
- Is a technically problematic method [23, 53, 75].

### cgMLST analysis

cgMLST was performed by assigning specific alleles to core genes. In 2018, researchers developed the cgMLST core genome typing scheme to distinguish and differentiate *Brucella* species into biovars using 407 genome sequences [75]. In the same year, other scientists developed a cgMLST scheme for *B. melitensis*, which included 2656 genes [53]. In 2022, researchers published the results of creating a cgMLST scheme applicable to all *Brucella* species, which included 1325 *Brucella* genomes [82].

cgMLST is the most advanced method, in contrast to MLST, as it no longer analyzes 21 gene loci but analyzes hundreds to thousands of significant genes [83]. Thus, cgMLST is superior to MLST in discriminative power. The method is expensive.

## MLVA

Epidemiologic monitoring using molecular genetic methods provides evidence of the geographic origin of pathogens.

To distinguish between species, biovar, and even isolates of highly conserved *Brucellae*, scientists have used MLVA since 2003. The publicly available MLVA database allows information about the causative agent of brucellosis to be entered and compared with the existing database [25]. MLVA is considered a tool similar to fingerprinting [84]. The MLVA method is regarded as the gold standard for *Brucella* typing, and it is based on the PCR method for differentiating strains of *Brucella* spp. and elucidates the causes of its occurrence and spread [29, 30, 73, 85]. Researchers have proposed MLVA15 methods: a gel-based MLVA technique, MLVA-15I Institute of Molecular Genetics (IGM), and an automated capillary electrophoresis-based method, MLVA-15 Northeast Agricultural University (NAU). The MLVA-15NAU assay detected more alleles and a higher diversity index than the markers examined in the MLVA-15IGM assay. Comparing the two methods, the MLVA-15NAU method is more expensive but more reproducible.

The 16 MLVA loci included moderately variable minisatellites (panel 1) and highly discriminatory microsatellites (panels 2A and 2B). More recently, in Kazakhstan, the genotypes of *Brucella* spp. were monitored using MLVA and compared with global isolates. In our previous study, we performed genotyping of circulating *Brucella* spp. in Kazakhstan, revealing that isolates from cattle, small ruminants, and humans belonged to the most common pathogens, *B. melitensis* biovar 1–3 (mainly biovar 1 and II genotypes). In addition, we found genetically unique isolates from cattle, small ruminants, and camels that belonged to *B. abortus* biovar 1, 2, 3, 5, and 6 (mainly biovar 3) [86].

The widespread dissemination of next-generation sequencing methods has made it possible to move from classical MLVA to MLVA based on WGS [17]. Chinese scientists studied *B. melitensis* strains using two methods: MLVA and WGS-SNP. Because of MLVA, all strains belonged to the Eastern Mediterranean lineage. WGS-SNP identified genotype II, which was divided into six subclades, four of which formed independent lineages, suggesting that local circulating lineages may increase the incidence of human brucellosis. Thus, the resolution of WGS-SNP exceeded that of MLVA [87].

### Advantages of the MLVA method

- Typing data are available online, it is easy to compare laboratories and countries, and it has good discriminatory power.

### The disadvantages of the MLVA method

- Convergent evolution, which makes it challenging to analyze phylogenetic relationships [9]
- MLVA examines only specific target regions of the genome, and tandem repeat markers are not informative enough
- In addition, SNPs (cgSNPs) have higher resolution than MLVA because SNPs across the entire bacterial genome are examined, unlike MLVA, in which only tandem repeat loci are analyzed [50, 53, 88]
- This method is expensive and requires a qualified researcher.

## Analysis of cgSNP based on WGS

### WGS

Researchers have found that several types have genetic differences, even in a single species of bacteria. DNA-DNA hybridization and comparative genomics have shown that *Brucella* species share >80% homology and >98% sequence similarity, which is one of the challenges in molecular epidemiology in species identification [36], primarily using molecular typing tests [89]. The 16S ribosomal RNA sequence was 100% identical between all *Brucella* spp.

To understand where the strains originated from and whether they are related, it is necessary to use various genetic tools, such as WGS [32, 50, 90–92]. WGS is a widely used tool for molecular typing and evolutionary studies [9, 53] and for studying geographic distribution and emerging pathogens [93]. WGS replaces the traditional molecular typing approach of pulsed-field gel electrophoresis (PFGE) and complements MLST [79, 80]. PFGE has high discriminatory power and typability, but the test requires 3–5 days to complete. The cost is relatively high compared with other methods, and the availability of this method is limited. The PFGE and amplified fragment length polymorphism methods provide different laboratory results.

Five different *Brucella* genotypes, as well as several sub-genotypes, have been identified using WGS technology [23]: The most basic lineage is the Western Mediterranean clade (genotype I); Eastern Mediterranean includes the Middle East (genotype II), African (genotype III), European (genotype IV), and American (genotype V) [22, 32]. It was previously reported that most Asian strains of *B. melitensis* belong to genotype II [86, 94]; thus, all Indian and Chinese strains of *B. melitensis* belong to the Eastern Mediterranean clade [25], whereas genotypes III, IV, and V have limited geographical distribution [24, 95]. The phylogenetic tree shows that isolates of *B. melitensis* species originated from the Mediterranean region [23, 42]. Thus, *B. melitensis* isolates spread across the Mediterranean Sea through livestock and animal products destined for trade [23].

WGS data represent a complete collection of genes and can differentiate the genetic features of closely related strains, even when conventional methods cannot identify differences between strains from the same outbreak or strains circulating in a specific geographic area [91] due to extremely high similarity between isolates [79, 92]. Recently, WGS has become simple, cost-effective, and accessible [23]. With the development of the WGS method, bioinformatics packages that can differentiate related pathogens are also evolving [23, 50, 53, 96, 97]. The availability of complete genome sequences in databases, e.g., microbial taxonomy data, allows for whole-genome comparative studies of different bacterial species to explore the presence of possible lineages in a region [92]. The National Center for Biotechnology Information (NCBI) database contains 355 strains of *B. melitensis* [98] and 175 strains of *B. abortus* [89]. Biovar, especially *B. melitensis*, is poorly correlated with certain genetic entities [23], and as such, there is a need to utilize molecular genetic testing methods. MLVA and MLST are currently used to classify the *Brucella* species into genus [99], and the availability of WGS in public databases allows comparisons of the genomes of different pathogens. At the same time, it remains difficult to determine the pathogen’s origin and possible migration route [50, 76].

Genotype classifications have been confirmed in WGS studies, for example, by comparing the complete genomes of 11 *B. melitensis* isolates from Russia with 87 *B. melitensis* isolates from the NCBI Genbank and a survey of 57 imported cases in Germany [22]. The Indian strain ADMAS-G1, *B. melitensis* Rev. 1 (ST7), and the reference strain *B. melitensis* 16M were assigned to the American lineage. The similarity of isolates from India and China is related to trade between Asian countries [23]. Egyptian *B. melitensis* strains have been assigned to the Mediterranean lineage, indicating their phylogenetic relationship with strains from the Mediterranean region [9]; a comparison of 13 NCBI *B. melitensis* genomes with the genomes of 25 *B. melitensis* isolates from patients in Norway [22] showed that most of them belong to the Eastern Mediterranean lineage and the remainder to the African lineage. Brucellosis is often detected in migrants and travelers returning from endemic countries [32]. The 27 Israeli *B. melitensis* strains were genotype II [22].

#### Advantages of the WGS method

- Fast and reliable [79, 92]
- Provides a pan-genomic assessment of *Brucella* genome variation, including virulence genes, and allows comparisons with databases of virulence factors and examination of how these genes differ between strains [22].

#### Disadvantages of the WGS method

- Expensive, requires special equipment, and qualified specialists.

### Analysis of cgSNP based on WGS

After WGS, it is necessary to identify SNPs among strains [100]. Initially, SNP analysis was based on real-time PCR. This method is a simple and rapid approach to identifying *Brucella* isolates at the species level. The recent introduction of SNP-based typing, which is associated with reduced costs, has significantly improved molecular subtypic and phylogenetic analysis in microbiology [17]. The cgSNP tracks brucellosis and facilitates accurate intraspecies differentiation and comparative analysis of *Brucella* isolates and biovar [50, 101, 102], providing sufficient data for comparison [3]. Although fewer genomes are available for comparative SNP studies than for MLVA allelic profiles, WGS-SNP analysis provides better resolution because polymorphism can be inferred based on coding and non-coding regions [50], including intergenic regions and covering more regions of the genome compared with MLST or core genome phylogeny [103]. In addition, an SNP microarray was previously used to infer the evolutionary lineage of *Brucella* spp. [104]. Core-genome SNP analysis is a reliable method for molecular genotyping [105].

The SNP method is suitable for genotyping all *Brucella* species. However, most authors describe SNP results for the *B. melitensis* species because this species is the most common and dangerous *Brucella* species for humans. Scientists have described the use of SNPs to effectively differentiate *B. melitensis* isolates and determine their geographic and worldwide distribution [50, 106]. The authors described that using the WGS-SNP method, related *B. melitensis* strains can be identified and better differentiated when isolates are formed into genotypes, depending on the circulation in a particular territory. Changes in *B. melitensis* genes allow them to adapt to new geographical areas and hosts [23, 107]. In this context, SNPs helped to provide insights into the evolution of *B. melitensis* strains. In addition, the cgSNP method can be used to detect *B. melitensis* isolates occurring in a specific geographical area.

Bioinformatics analysis has been used to process raw molecular typing data to elucidate biological processes [108]. For meaningful WGS-SNP analysis, errors encountered during data preparation, amplification, software, sequencing, and sequence mapping/alignment must be considered [109]. In addition, when performing WGS-SNP, using a reference strain is essential because it determines the reliability of the results in establishing relationships between isolates. At present, researchers use the reference strains *B. abortus* 2308 and *B. melitensis* 16 M [48, 53]. SNP calling requires well-processed data and a user-friendly and reliable calling tool [89].

WGS-SNP analysis is a rapid tool for epidemiological studies because it can effectively distinguish 33 *Brucella* isolates by determining their geographic origin [110]. This method is more efficient than MLVA and cgMLST, but less variation was observed [89]. The WGS-SNP method revealed that *B. melitensis* isolates from India were similar to the vaccine strain *B*. *melitensis* M5 from China and had no similarity to the vaccine strain *B*. *melitensis* Rev.1 [97]. cgSNP analysis in Iran revealed similarities between *B. abortus* isolates and strains from neighboring and Middle Eastern countries. In Ethiopia, phylogenetic analysis based on the wgSNP revealed that *B. abortus* belongs to lineage A [88]. The isolates from Iran *B. melitensis* investigated using the cgSNP method were classified as American and Eastern Mediterranean clades [95].

#### Advantages of the cgSNP method

- Fast, reliable, and reproducible [53]
- High resolution [103].

#### The disadvantages of the cgSNP method

- Expensive, requires special equipment, and qualified specialists.

## Discussion

Brucellosis, a zoonotic disease, harms livestock productivity and endangers human health. Brucellosis spreads due to the international exchange and transportation of infectious livestock without control. Thus, *B. melitensis* isolates have spread across the Mediterranean Sea with livestock and animal products destined for trade [23], whereas *B. abortus* isolates have spread from South Africa due to socioeconomic, migration, and colonial relations among countries [74]. Sharing pastures with neighboring livestock increases the risk of brucellosis infection. Across the country, open and mixed animal markets contribute to the dissemination of brucellosis. Most farmers disregard the rules against brucellosis, making their herds vulnerable by acquiring replacement stock without verifying their disease status.

Activities that can help eliminate brucellosis include detailed epidemiological studies and genotyping of circulating strains to identify and determine the sources and routes of infection. To prevent brucellosis, it is necessary to import animals only from countries known to be free of the disease. It is crucial to consistently educate about the harmful effects of vaccine misuse and the illicit transfer of livestock among herds. Coordinated efforts among the ministries of health, agriculture, the environment, and natural resources are essential for enhancing brucellosis surveillance and control and for ensuring optimal health for humans, animals, and the ecosystem. Enhancing collaboration between farmers and the government is essential for fostering robust veterinary services.

Social factors, such as trade, migration, and travel, impact the transmission of brucellosis across international borders. The country’s economic potential is substantial due to the requirement of substantial resources to eradicate the disease, including expensive equipment and reagents, trained personnel, and reasonable compensation for animals. Farmers, veterinarians, and scientists receive full support from state policy. The ecological condition of a country can influence the spread of pathogens among wild animals. Implementing organizational, economical, unique, and sanitary measures along with allocating necessary resources is crucial in preventing brucellosis in animals and eradicating its sources.

A definitive diagnosis of brucellosis in animals requires a combination of serological reactions, culture isolation, and biochemical tests. Based on the current epidemiological context, rose Bengal test (RBT) and complement fixation test (CFT) are used for animal diagnosis. In cases of doubtful results, the standard tube-agglutination test served to clarify the animals’ status. Animals are sent for slaughter if they test positive for disease in PCR results or culture isolates. Eradicating brucellosis using these methods is challenging due to the inability to trace the source and transmission pathway of the infectious agent. These methods should provide reliable, reproducible, and discriminatory results. Scientists are refining new molecular genetic techniques. The WGS method enables accurate identification of strains’ geographical origin and distribution. Identifying the infection source can contain the causative agent and halt the epidemiological process early on.

The genus *Brucella* includes 14 species that differ in biochemical characteristics, host preference, and degree of pathogenicity. The control of brucellosis mainly depends on the efficiency of detecting and analyzing the predominant *Brucella* species in a particular area. Using WGS and MLVA, it is possible to determine the geographic origins of strains and therefore the source of pathogens. However, MLVA has limitations because only tandem repeat loci are examined. The WGS method has more advantages than MLVA because it can better discriminate pathogen genotypes [32, 53, 111]. Because some strains circulate only in certain areas, it is possible to trace pathogen introduction through genetic changes in isolates [50]. WGS was used to identify patterns of brucellosis emergence in individual countries and the spread of the disease within a country [94]. WGS provides an opportunity to clarify the geographic origin of an isolate or outbreak when information about the likely country of infection is missing or unclear [32].

The WGS method boasts superior resolution compared to other molecular typing methods. The development of WGS technology allows for the comprehensive analysis of *Brucella* genome variations, thereby aiding in the elimination and prevention of brucellosis. Despite its speed and reproducibility, the WGS method is not accessible to all researchers due to financial constraints.

Incorporating advanced molecular techniques into conventional methods for pathogen identification and elimination is necessary due to limitations in WGS.

## Conclusion

Based on the literature, we present a brief overview of methods for investigating *Brucella*. We emphasized molecular genetic methods for *Brucella* research. Molecular genetic typing techniques for pathogens have advanced over the last few decades. The use of each molecular genotyping method depends on an institution’s resources, personnel training, and purpose.

*Brucella* typing is particularly difficult due to the extreme likeness among isolates. Based on our molecular genetic analysis, we recommend WGS for characterizing *Brucella* strains. The WGS method has surpassed the MLVA method as the gold standard for *Brucella* genotyping due to its greater power. The WGS method identifies the precise geographical origin and distribution routes of strains. WGS offers superior genotype distinction compared to other methods due to its comprehensive genome coverage. WGS has recently been simplified, cost-effective, and made more accessible.

## Authors’ Contributions

AD: Conceptualization and writing-original draft. SD and SP: Reviewed and edited the manuscript. NK: Designed the study. All authors have read, reviewed, and approved the final manuscript.
